# Personality and Personality Disorders in Medication-Overuse Headache: A Controlled Study by SWAP-200

**DOI:** 10.1155/2019/1874078

**Published:** 2019-06-12

**Authors:** Federica Galli, Annalisa Tanzilli, Alessandra Simonelli, Cristina Tassorelli, Grazia Sances, Micol Parolin, Patrizia Cristofalo, Ivan Gualco, Vittorio Lingiardi

**Affiliations:** ^1^ASST SS. Paolo and Carlo, S. Paolo Hospital, Milan, Italy; ^2^Department of Dynamic and Clinical Psychology, Faculty of Medicine and Psychology, Sapienza University of Rome, Rome, Italy; ^3^Department of Developmental Psychology and Socialization, University of Padova, Padova, Italy; ^4^Department of Brain and Behavioral Sciences, University of Pavia, Pavia, Italy; ^5^Headache Science Center, IRCCS Mondino Foundation, Pavia, Italy; ^6^Therapeutic Community “Villa Renata”, Venice, Italy; ^7^Center for Individual and Couple Therapy, Genoa, Italy

## Abstract

**Background:**

Medication-overuse headache (MOH) is a type of chronic headache, whose mechanisms are still unknown. The impact of psychological factors has been matter of debate from different perspectives. The role of personality and personality pathology in processes involved in MOH development has been advanced but was poorly studied. The hypothesis of addiction-like behaviors sustaining the drug misuse has been examined and reached contrasting findings.

**Objectives:**

This study is aimed at detecting personality and its disorders (PDs) in MOH, with a specific attention to the addiction aspect.

**Methods:**

Eighty-eight MOH patients have been compared with two clinical populations including 99 patients with substance use disorder (SUD) and 91 with PDs using the Shedler-Westen Assessment Procedure-200 (SWAP-200), a clinician-report tool that assesses both normal and pathological personality. MANCOVAs were performed to evaluate personality differences among MOH, SUD, and PD groups, controlling for age and gender.

**Results:**

MOH patients were predominantly women and older. They showed lower traits of the SWAP-200's cluster A and B disorders than SUD and PD patients, who presented more severe levels of personality impairment. No differences in the SWAP-200's cluster C have been found, indicating common personality features in these populations. At levels of specific PDs, MOH patients showed higher obsessive and dysphoric traits and better overall psychological functioning than SUD and PD patients.

**Conclusion:**

Although MOH, SUD, and PD populations have been evaluated in multiple sites with different levels of expertise, the study supported the presence of a specific constellation of personality in MOH patients including obsessive (perfectionist) and dysphoric characteristics, as well as good enough psychological resources. No similarities to drug-addicted and personality-disordered patients were found. Practitioners' careful understanding of the personality characteristics of MOH patients may be useful to provide a road map for the implementation of more effective treatment strategies and intervention programs.

## 1. Introduction

Medication-overuse headache (MOH) is a type of chronic headache associated with the overuse of one or several forms of acute painkilling treatments and a consequent worsening of a preexisting headache. First described in the 1980s [1], this disease is included in Third Edition of the International Classification of Headache Disorders (ICHD-3), [2], even if the scientific debate on the nosology, definitions of overuse, and pathophysiological mechanisms is still ongoing [3]. MOH is a worldwide problem, with prevalence rates ranging between 1 and 2%, most commonly among women between 40 and 50 years of age [4]. MOH represents 55–70% of the population that consults headache centers [5].

Although the progressively increasing use of acute medications is widely considered the most important factor for transforming episodic headaches into MOH [6, 7], the role of psychological factors has also been underscored in many empirical investigations, mainly in terms of psychopathology (especially anxiety and mood disorders [8], with psychiatric comorbidity representing a well-known negative prognostic factor [9–11].

Antecedent anxiety and depression have been suggested to have a crucial role in the development of MOH [12]. However, after almost 30 years since the first studies on psychiatric disorders in conjunction with headaches [13], it is not possible to go beyond the simple description of a comorbid association with (mostly) anxiety and/or mood disorders [14]. Thus, it is important to scrutinize the generic concept of “psychiatric comorbidity” by studying personality and individual psychological factors other than psychiatric ones. Although Wolf's description of a “migraine personality” (i.e., ambitiousness, extreme tidiness, perfectionism, inflexibility, and resentment) dates back to 1937 [15], research on personality and headache is scarce and inconclusive. From this perspective, studying personality and its disorders (PDs) may be fruitful as it takes into account that a growing body of literature supports a robust association of personality pathology and health problems [16, 17]. The attempt to depict a pain-prone personality is ongoing [17], with higher avoidance harm and lower self-directedness (assessed by the Temperament and Character Inventory [18]), as the most distinguishing candidate personality features of chronic pain patients. Harm avoidance and self-directedness have been examined in headache but contrasting research findings have been obtained [19–22]. The presence of personality pathology has been linked more frequently to chronic, rather than episodic headache [23]. Bigal et al. [24] have used the clinical scales of the Minnesota Multiphasic Personality Inventory-2 (MMPI-2) [25] and found no difference between MOH and chronic migraine, compared to migraine and new daily persistent headache. Conversely, some research has been conducted using the Structured Clinical Interview for DSM-IV Personality Disorders (SCID-II) [26] and considered the DSM [27, 28] classification of three PD clusters (cluster A includes schizoid, paranoid, and schizotypal disorders, characterized by odd or eccentric features; cluster B includes antisocial, borderline, narcissistic, and histrionic disorders, characterized by dramatic and impulsive patterns of behavior; and cluster C includes avoidant, dependent, and obsessive-compulsive disorders, characterized by anxious or fearful patterns of behavior). These studies have found that chronic migraine was associated with an overall prevalence of about 80% for any personality disorder [29, 30], with obsessive-compulsive personality disorder as the most prevalent. Interestingly, a study on physical comorbidity in patients with PDs evidenced the prevalence of cluster C (avoidant, dependent, and obsessive) disorders in conjunction with recurrent headache [31].

From the MOH aspect, some studies focused on dependence from drugs as the psychological mechanism supporting medication overuse. Some authors suggested a link between MOH patients and those with addiction spectrum disorders [32, 33]. Genetic research to appraise gene polymorphism association in MOH and detect genes related to drug dependence pathways in the MOH population did not produce any definitive conclusion [34]. Neuroimaging studies on MOH found abnormalities in cerebral regions linked to dependence and addiction [35, 36]. Studies that assessed personality using the MMPI-2 highlighted a completely different personality configuration compared to patients with drug addiction [37]. It must be noted that the use of the MMPI-2 has been criticized because many items use somatic symptoms to assess underlying traits [17, 38], which may confound the assessment among chronic pain sufferers. Hence, there is a need to identify new assessment tools, rather than base evaluations on self-report measures that may suffer from a lack of sufficient criterion validity (e.g., [39]).

The main aim of the present study was to examine personality and its disorders in patients with MOH using a clinician-report personality measure, the Shedler-Westen Assessment Procedure-200 (SWAP-200), [40, 41]. We performed an exploratory analysis of personality characteristics within a group of MOH patients that have been compared to a group of patients with addiction and a group of patients with PDs.

## 2. Materials and Methods

### 2.1. Participant Sampling

The population samples analyzed in the present study were recruited in diverse centers within the Italian National Health System that specialize in the treatment of clinical populations with 3 three forms of diseases. A team of expert practitioners were directed to select (a) a group of chronic headache patients (MOH) enrolled at the IRCCS “C. Mondino National Institute of Neurology Foundation” in Pavia; (b) a group of patients with the DSM-5 [28] substance use disorder (SUD) enrolled at the therapeutic community “Villa Renata” in Venice; and (c) a group of patients with PDs enrolled at Italian psychological associations for the treatment of personality pathology in Rome, Genoa, Milan, and Turin. According to the inclusion criteria of the study, these patients were at least 18 years old, had no psychotic disorder or syndromes with psychotic symptoms, and had no mental retardation or clinically relevant cognitive impairment.

Practitioners' assessment is the source of data used in this empirical investigation. A neurologist, clinical psychologists, and psychiatrists were asked to conduct three or four clinical interviews and yield accurate information regarding the patients who met the study's criteria. They also completed a comprehensive diagnostic assessment procedure to assess patients' personality disorders and psychological functioning. All participants provided written informed consent. The study protocol received ethics approval from the local research ethics review board.

### 2.2. Practitioners

The sample consisted of 1 neurologist, 15 clinical psychologists, and 5 psychiatrists (*N*=21). Thirteen were women and 8 were men. The mean age of all practitioners who rated patients by SWAP-200 was 43 years (SD = 5.37, range = 33–52). The average length of their clinical experience was 14 years (SD = 5.20, range = 4–20). The main clinical-theoretical orientation of psychologists/psychiatrists was psychodynamic (*N*=19); only one clinical psychologist had a systemic family approach. All assessors had received the same formal training in the use of SWAP-200.

### 2.3. Patients

Our population consisted of 278 Caucasian patients who were subdivided in the following samples.

#### 2.3.1. Medication-Overuse Headache Group

This group consisted of 88 consecutive in-patient MOH, diagnosed according to the ICHD-III beta criteria [2]. Sixty-seven were women and 21 were men. Their mean age was 46.88 (SD = 9.97, range 19–64). The patients were diagnosed by a neurologist (GS) who collected clinical information on headache and sociodemographic data, along with the history of present and previous use of medications and/or other substances. The same neurologist verified the eligibility criteria. The mean duration of chronic headache was 6.1 years (range: 5 months–29 years), and the mean duration of symptomatic drug overuse was 4.7 years (range: 6 months–28 years). On the basis of the data contained in the headache diaries, we recorded an average of 23 headache days (range: 15–30), 22 days of symptomatic drug intake (range: 10–30), and 39 doses taken monthly (range: 10–220).

#### 2.3.2. Substance Use Disorder Group

This group consisted of 99 patients, diagnosed according to the DSM-5 criteria (present/absent) for substance-related and addictive disorders. Fifty-seven were women and 42 were men. Their mean age was 22.89 (SD = 4.62, range 18–45). The majority of them indicated heroin as the primary substance of abuse. The assessment took place, on average, 1.6 months after the patients' admission. At the time of recruitment, the patients had abstained from drugs for an average of 3 months.

#### 2.3.3. Personality Disorder Group

This group consisted of 91 patients, diagnosed according to the PD criteria (present/absent) of the DSM-5 classification system. Forty-five were women, and 46 were men. Their mean age was 36.88 (SD = 11.20, range 20–65). Sixteen had cluster A diagnoses (including paranoid, schizoid, and schizotypal disorders), 29 had cluster B diagnoses (including antisocial, borderline, histrionic, and narcissistic disorders), and 46 had cluster C diagnoses (including avoidant, dependent, and obsessive-compulsive disorders). They had no comorbid SUD. Seventy-five percent of the patients were from private practice, and the remaining 25% were from public mental health institutions.

### 2.4. Measures

#### 2.4.1. Clinical Questionnaire

We used a questionnaire for the neurologist, clinical psychologists, and psychiatrists to collect comparable general information from the different patient populations. The patients' sociodemographic data, age at onset of the disorder, and drug consumption were collected. Moreover, this questionnaire gathered general information on all practitioners (such as gender, age, years of experience, training, and clinical orientation).

#### 2.4.2. Shedler-Westen Assessment Procedure-200

The Shedler-Westen Assessment Procedure-200 (SWAP–200) [40–44] is a validated and reliable instrument designed to provide a comprehensive assessment of patient personality and psychological functioning based on the quantification of observations from therapists or clinical observers. This Q-sort instrument consists of a set of 200 personality-descriptive statements, written in jargon-free language near to clinical experience, to be used by practitioners with varying theoretical orientations and levels of experience. The assessor arranges these 200 statements into eight different categories ranging from 0 (*irrelevant or not descriptive of the person*) to 7 (*most descriptive*). Based on the Q-sort method [45], the SWAP–200 requires the assessor to assign a specified number of items to each score category (8 items in pile 7; 10 items in pile 6; 12 items in pile 5, etc.) in order to comply with the fixed distribution. The SWAP–200 assessment provides (a) a personality diagnosis expressed as the matching of the patient assessment with 10 personality disorder scales, which are clinical prototypes of the DSM-IV and DSM-5 [27, 28] personality disorders (PD scales) and (b) a personality diagnosis based on the correlation/matching of the patient's SWAP description with 11 styles/syndromes of personality derived empirically via Q-factor analysis (Q-factors). It also includes a dimensional measure of psychological strengths and adaptive functioning. All SWAP–200 PD scales and Q-factors make it possible to obtain both categorical and dimensional diagnoses. In further detail, the presence of one or more personality disorders is established when one or more PD scale and/or Q-factor score (in standardized *T* points) is ≥60 and the score on the high-functioning scale is ≤60; if the score ranges from 55 to 60, then the subclinical traits of that personality disorder or style are present. The SWAP-200 has been extensively shown to have very good validity and reliability in several studies conducted on different clinical populations (e.g., [46–49]).

### 2.5. Statistical Analysis

Statistical analyses were performed using SPSS 20 for Windows (IBM, Armonk, NY). The chi-square test and analysis of variance (ANOVA) were carried out to explore differences among MOH, SUD, and PD patient groups on gender and age, respectively. Then, a series of multiple analyses of covariance (MANCOVAs) with Bonferroni post hoc analyses (*p* < 0.05) were performed to assess MOH, SUD, and PD group differences on patients' personality disorders and psychological functioning (assessed using the SWAP-200) while controlling for gender and age as covariates. In the first MANCOVA, the data on patients' personality pathology were analyzed at the PD cluster level (by aggregating the SWAP-200 paranoid, schizoid, and schizotypal PD scales for cluster A; the SWAP-200 antisocial, borderline, histrionic, and narcissistic PD scales for cluster B; and the SWAP-200 avoidant, dependent, and obsessive-compulsive PD scales for cluster C). Further, for each patient, the average scores of the SWAP-200 PD scales that comprised each cluster were calculated. Conversely, in the second and third MANCOVAs, the data were analyzed at the single-disorder level by using the SWAP-200 PD scales and Q-factors, respectively.

## 3. Results

### 3.1. Differences among MOH, SUD, and PD Patient Groups on Demographic Characteristics

First, MOH, SUD, and PD patient groups were compared on gender and age. As expected, there were significant differences on these two demographic variables. The three patient groups differed on gender (*χ*2 (2) = 13.16, *p*=0.001). Men were more likely to be classified as SUD (38.9% of men versus 33.5% of women) and PD patients (41.7% of men versus 27.1% of women), while women were more likely to be classified as MOH patients (19.4% of men versus 39.4% of women). Moreover, the patient groups significantly differed on age, *F*(2,275) = 170.72, *p* < 0.001, *η*^2^ = 0.55. The ANOVA's results revealed that SUD patients (*M* = 22.89) were younger than PD patients (*M* = 37.01) and PD patients were younger than MOH patients (*M* = 46.88).

### 3.2. Differences among MOH, SUD, and PD Patient Groups on Personality Pathology and Psychological Functioning

The main aim of the study was to compare the MOH, SUD, and PD patient groups on personality pathology (at the level of PD clusters) and psychological functioning (evaluated using the SWAP-200) while controlling for the effects of gender and age. A first MANCOVA was performed using patient groups as the independent variable, the three clusters of the SWAP-200 PD scales as dependent variables, and gender and age as covariates. The results revealed significant main effects for the groups (Wilks's *λ* = 0.70, *F*(6,542) = 17.47, *p* < 0.001, *η*^2^ = 0.16), while no significant effect was found for gender (Wilks's *λ* = 0.99, *F*(3,271) = 0.83, *p*=0.48, *η*^2^ = 0.01) and age (Wilks's *λ* = 0.98, *F*(3,271) = 1.62, *p*=0.19, *η*^2^ = 0.02). Follow-up univariate analyses with Bonferroni post hoc tests (*p* < 0.05) indicated that all three patient groups significantly differed on the SWAP-200's clusters A and B, while no difference was revealed on cluster C (Table 1). In particular, SUD patients showed higher mean scores of clusters A and B as compared to those obtained by the PD and MOH patients.

The second MANCOVA was conducted to investigate the differences among the MOH, SUD, and PD patient groups on personality disorders and global psychological functioning (assessed using the SWAP-200 PD and high-functioning scales) while controlling for the effects of gender and age (as covariates). The results showed that the gender had a significant effect on personality variables (Wilks's *λ* = 0.90, *F*(11, 263) = 2.62, *p* < 0.01, *η*^2^ = 0.09), while no effect of age was found (Wilks's *λ* = 0.94, *F*(11, 263) = 1.43, *p*=0.16, *η*^2^ = 0.06). The MANCOVA's findings revealed that even after adjusting for covariates, there were significant effects for the groups on the SWAP-200 PD and high-functioning scales (Wilks's *λ* = 0.49, *F*(22, 526) = 10.28, *p* < 0.001, *η*^2^ = 0.30). Further, the post hoc analyses by Bonferroni's correction showed significant differences among the MOH, SUD, and PD patient groups on all SWAP-200 PD scales, with the exception of the schizoid and avoidant personality disorders (Table 2). MOH patients had significantly lower scores in the SWAP-200 paranoid, schizotypal, antisocial, borderline, histrionic, narcissistic, and dependent PD scales and higher scores in the SWAP-200 obsessive PD and high-functioning scales than those obtained by SUD and PD patients.

Finally, the last MANCOVA was conducted to examine the differences among the MOH, SUD, and PD patient groups on personality styles/syndromes derived empirically from the SWAP-200 (Q-factors), while controlling for the effects of gender and age (as covariates). The findings revealed that gender had a significant effect on personality variables (Wilks's *λ* = 0.88, *F*(11, 263) = 3.22, *p* < 0.001, *η*^2^ = 0.12), while age did not show any effect (Wilks's *λ* = 0.95, *F*(11, 263) = 1.38, *p*=0.18, *η*^2^ = 0.05). The MANCOVA's results demonstrated that even after adjusting for covariates, there were significant effects for the groups on the SWAP-200 Q-factors (Wilks's *λ* = 0.40, *F*(22, 526) = 13.87, *p* < 0.001, *η*^2^ = 0.37). Moreover, the post hoc analyses by Bonferroni's correction showed significant differences among MOH, SUD, and PD patient groups on all SWAP-200 Q-factors, with the exception of the paranoid, schizoid, and dysphoric: avoidant personality styles/syndromes (Table 3). MOH patients had significantly lower scores in the SWAP-200 antisocial, histrionic, narcissistic, dysphoric: emotionally dysregulated, dysphoric: dependent-masochistic, and dysphoric: hostile-externalizing Q-factors and higher scores in the SWAP-200 obsessive PD and DS: high-functioning neurotic Q-factors than those obtained by SUD and PD patients.

## 4. Discussion

The present study sought to investigate personality characteristics and psychological functioning in a clinical population with MOH using the SWAP-200, a valid and reliable clinician-report instrument. A group of patients with this kind of chronic headache was compared on specific individual variables (gender and age) and personality dimensions to two different clinical groups including patients with SUD and PDs, respectively.

Overall, the results showed that MOH is most prevalent in women and older patients, thus confirming previous research (e.g., [4, 50]). Moreover, the study indicated that distinct personality traits distinguish MOH from SUD and PD patients, regardless of demographic characteristics, in a clinically meaningful manner. The findings demonstrated significant differences at the level of SWAP-200 PD clusters A and B among these clinical populations. MOH patients presented low traits of personality syndromes characterized by affective flattening, interpersonal deficits in close relationships, odd behaviors and eccentric and idiosyncratic reasoning processes or beliefs, or by impulsivity and emotional dysregulation, severe impairments in interactions with others, and identity and behavior disturbances. Conversely, there were no differences among MOH, SUD, and PD patients at the cluster C level. These results elucidate a consistent overlapping of anxious traits in these populations and, especially, support the data of empirical studies, showing a strong association among patients with recurrent headache and avoidant, dependent, and obsessive-compulsive PDs [31].

Looking at the specific and nuanced results in Tables 2 and 3, MOH patients seem to show a specific personality configuration including obsessive (perfectionist) and dysphoric features that is completely different from the configuration of the SUD group. Notably, these results support previous research using the MMPI-2 [37, 51]. In detail, at the level of the SWAP-200 PD scales (Table 2), MOH patients presented a personality and psychological functioning that is different from that of SUD and PD patients and is characterized by the highest obsessive traits and the lowest paranoid, schizotypal, antisocial, borderline, histrionic, narcissistic, and dependent characteristics. These results were partially confirmed in terms of personality styles or SWAP-200 Q-factors (Table 3). Obsessive and dysphoric/high functioning neurotic traits were the most representative features of MOH patients as compared to SUD and PD groups, which were mostly characterized by histrionic, antisocial, dysphoric/dependent-masochist, dysphoric/emotionally dysregulated, and dysphoric/hostile-externalizing features.

Interestingly, in our study, borderline personality characteristics (in terms of SWAP-200 PD scales) are poorly represented in MOH patients, despite a study that showed an increased risk of developing MOH when migraine is comorbid with a borderline personality disorder (BPD) [52]. These findings were confirmed in terms of Q-factors of the SWAP-200, given that chronic headache patients did not present any personality style (histrionic, dysphoric: emotionally dysregulated, or dysphoric: dependent-masochistic Q-factors) that is typically linked to BPD [41]. This aspect deserves further attention because epidemiological research shows that individuals who screened positive for BPD had a high prevalence rate of chronic pain (19%) and a 12-month headache rated in 42% of patients with BPD [53].

From the side of the SUD group, the role of borderline (and antisocial) personality disorder (cluster B) is a clear-cut finding that is already recognized in the literature [54]. Obviously, this aspect further supports the psychological differences of MOH and drug addiction in the likely behavioral mechanisms supporting drug misuse. The psychological dimensions featuring MOH patients seem to be more related to the side of obsessiveness, bearing in mind that it is distinct from the obsessive-compulsive disorder [40, 41]. The study of personality features may be a key to explaining the route to medication overuse that, we hypothesized, is very different from a simple addiction to analgesic drugs [55] or “obsessive-compulsive disturbances for abused drugs” [56]. The prototypic description of obsessive personality [40, 41] refers to “patients excessively devoted to work and productivity, to the detriment of leisure and relationships… with difficulty acknowledging or expressing anger… self-critical, tending to set unrealistically high standards for themselves, showing little tolerance for their own human defects, and expecting themselves to be “perfect.” These individuals may adhere rigidly to daily routine and become anxious and uncomfortable when they are altered.” In this psychological framework, analgesics might become a necessary crutch with which to cope with life demands in spite of recurrent pain and not a way to seek pleasure or to escape from reality as may occur in addiction. Finding a genetic explanation for such specific behavior (drug misuse in chronic headache patients) is intriguing [34], but many aspects need to be taken into consideration. Addiction, as in many behaviors affecting health with negative outcomes, is the result of genetic and environmental variables. Twin studies have established that the heritability, or the proportion of the variation in the population trait of addiction, ranges between 40% and 70% [57]. These data leave a considerable margin to environmental influences. Recently, it has been outlined that the beginning of drug taking behavior is more under environmental influences, while the progression to addiction seems to be associated with genetic influences [58]. Coping with a recurrent painful condition, often from infancy or adolescence (MOH patients had a long-lasting history of chronic pain), may be very challenging and the “dependence” from pain relief may pass through excessive drug intake. In our opinion, this psychological (or behavioral) mechanism is far removed from that substance addiction. SUDs have been theoretically, for example, by the self-medication theory [59], and empirically (e.g., [60]) linked to emotional suffering, rather than physical pain; drugs are used as a coping mechanisms in the attempt to relieve or change a range of under-regulated and overwhelming painful affect states, often related to premorbid and co-existing mental health disorders, such as mood, anxiety, and posttraumatic stress disorders [61–63].

A final note on the psychological health index defined by the SWAP-200 high-functioning scale is clinically relevant. This index assesses the resemblance or match between the patient and an ideal prototype representing optimal psychological health [40, 41] and serves as a global measure of personality functioning. Interestingly, MOH patients scored very high on this scale compared to both SUD and PD patients. The results suggested that patients with MOH show significant psychological resources and strengths in the milieu of an obsessive and dysphoric personality, while patients with SUD and PDs present globally more severe levels of psychological impairment. This aspect strongly supports the potential positive role of psychological interventions, both as a psychoeducational (for preventing drug misuse) and psychotherapeutic one. When working with specific clinical populations, such as the MOH patient group, practitioners should consider personality characteristics able to moderate treatment outcomes [64, 65].

Our study is not free of limitations. First, all patients were enrolled from clinical settings and we are not sure that they are representative of patient population with MOH, SUD, and PDs (Berkson's bias [66]). Future studies might enroll a patient group from the general community to compare personality characteristics in the MOH population and extend the generalizability of the study's findings. Moreover, the stability of some results over time should be verified by longitudinal research. Secondly, MOH patients were interviewed as inpatients, while SUD and PD samples were evaluated in the outpatient setting. Further empirical investigations in this area should seek to address these issues by involving diverse and wide patient samples, taking into account the distribution of the severity of pathology and various clinical conditions. Furthermore, the different populations were evaluated in multiple sites with different expertise. We attempted to control for the possible confounding effects (e.g., a common questionnaire for data recording and training for SWAP administration and interpretation) to the best of our abilities. Finally, demographic differences among the groups might have partially influenced the results that we observed, although we have adjusted for these specific variables in all of the analyses and the effect size (*η*^2^) estimations were mostly of a moderate or large magnitude [67].

In summary, this study supports the presence of a specific constellation of personality in MOH patients that included obsessive (perfectionist) and dysphoric traits, as well as good enough psychological resources. No similarities with drug-addicted and personality-disordered patients were found. In particular, substantial differences between MOH and SUD patients seem to confirm the results of previous research [37, 51]. Overall, these findings may be useful in providing a road map for the implementation of effective treatment strategies and intervention programs among this clinical population with chronic headache.

## Figures and Tables

**Table 1:** Differences among patient groups on the three clusters of SWAP-200 PD scales while controlling for gender and age.

SWAP-200	MOH group (*n*=88)		SUD group (*n*=99)		PD group (*n*=91)		*F*(2, 273)	*η* ^2^
	*M*	SD	*M*	SD	*M*	SD		
Cluster A	43.98^a^	0.77	49.86^b^	0.75	46.30^c^	0.61	11.06^*∗∗∗*^	0.08
Cluster B	41.76^a^	0.83	54.89^b^	0.80	48.42^c^	0.66	47.58^*∗∗∗*^	0.26
Cluster C	49.61	0.81	47.70	0.79	48.32	0.65	1.14	0.01

*Note*. MOH group = medication-overuse headache group; SUD group = substance use disorder group; PD group = personality disorder group; SWAP-200 = Shedler-Westen Assessment Procedure-200; *η*^2^ = measure of effect size in analysis of covariance. Alphabetical superscripts indicate significant differences in post hoc analyses. Means with different alphabetic superscripts (a, b, and c) were statistically significant, while means with identical alphabetic superscripts were found not to be significantly different; ^*∗∗∗*^*p* < 0.001.

**Table 2:** Differences among patient groups on the SWAP-200 personality dimensions and psychological functioning while controlling for gender and age.

SWAP-200 PD scales	MOH group (*n*=88)		SUD group (*n*=99)		PD group (*n*=91)		*F*(2, 273)	*η* ^2^
	*M*	SD	*M*	SD	*M*	SD		
Paranoid	41.57^a^	1.05	50.72^b^	1.01	45.92^c^	0.83	14.29^*∗∗∗*^	0.10
Schizoid	46.79	0.96	47.88	0.93	46.96	0.77	0.31	0.00
Schizotypal	43.59^a^	0.95	50.97^b^	0.92	46.03^c^	0.75	12.10^*∗∗∗*^	0.08
Antisocial	43.20^a^	0.86	53.56^b^	0.83	47.69^c^	0.68	27.67^*∗∗∗*^	0.17
Borderline	38.71^a^	1.10	56.60^b^	1.07	47.52^c^	0.88	49.44^*∗∗∗*^	0.27
Histrionic	42.11^a^	1.07	56.40^b^	1.03	50.03^c^	0.85	34.34^*∗∗∗*^	0.20
Narcissistic	43.00^a^	0.95	53.01^b^	0.92	48.45^c^	0.76	20.99^*∗∗∗*^	0.13
Avoidant	47.69	0.95	46.82	0.92	46.62	0.76	0.41	0.00
Dependent	47.43^a^	1.06	52.08^b^	1.02	49.10^a,b^	0.84	4.04^*∗*^	0.03
Obsessive	54.03^a^	1.00	44.15^b^	0.96	48.96^c^	0.80	17.62^*∗∗∗*^	0.11
High-functioning	63.05^a^	1.09	45.38^b^	1.05	54.89^c^	0.87	49.29^*∗∗∗*^	0.27

*Note*. MOH group = medication-overuse headache group; SUD group = substance use disorder group; PD group = personality disorder group; SWAP-200 = Shedler-Westen Assessment Procedure-200; *η*^2^ = measure of effect size in analysis of covariance. Alphabetical superscripts indicate significant differences in post hoc analyses. Means with different alphabetic superscripts (a, b, and c) were statistically significant, while means with identical alphabetic superscripts were found not to be significantly different; ^*∗*^*p* < 0.05; ^*∗∗∗*^*p* < 0.001.

**Table 3:** Differences among patient groups on the SWAP-200 personality styles/syndromes while controlling for gender and age.

SWAP-200 Q-factors	MOH group (*n*=88)		SUD group (*n*=99)		PD group (*n*=91)		*F*(2, 273)	*η* ^2^
	*M*	SD	*M*	SD	*M*	SD		
Antisocial	43.36^a^	0.85	53.94^b^	0.82	47.86^c^	0.67	29.64^*∗∗∗*^	0.18
Schizoid	46.42	0.96	48.18	0.93	46.69	0.77	0.80	0.01
Paranoid	48.40	3.38	49.15	3.27	47.02	2.69	0.15	0.00
Obsessive	60.21^a^	1.14	44.90^b^	1.10	53.96^c^	0.91	34.40^*∗∗∗*^	0.20
Histrionic	48.70^a^	1.05	54.09^b^	1.01	52.48^b^	0.83	5.70^*∗∗*^	0.04
Narcissistic	41.10^a^	1.03	48.65^b^	0.99	45.47^c^	0.82	17.27^*∗∗∗*^	0.11
DS: avoidant	49.86	0.95	46.69	0.92	47.54	0.76	2.47	0.02
DS: high-functioning neurotic	58.38^a^	0.98	47.13^b^	0.95	53.90^c^	0.78	25.20^*∗∗∗*^	0.16
DS: emotionally dysregulated	41.92^a^	1.05	51.89^b^	1.02	46.16^c^	0.84	16.96^*∗∗∗*^	0.11
DS: dependent-masochistic	41.42^a^	1.06	57.45^b^	1.03	49.62^c^	0.84	43.00^*∗∗∗*^	0.24
DS: hostile-externalizing	43.95^a^	1.06	50.68^b^	1.02	47.52^a^	0.84	7.65^*∗∗*^	0.05

*Note*. MOH group = medication-overuse headache group; SUD group = substance use disorder group; PD group = personality disorder group; SWAP-200 = Shedler-Westen Assessment Procedure-200; DS = dysphoric subfactor; *η*^2^ = measure of effect size in analysis of covariance. Alphabetical superscripts indicate significant differences in post hoc analyses. Means with different alphabetic superscripts (a, b, and c) were statistically significant, while means with identical alphabetic superscripts were found not to be significantly different; ^*∗∗*^*p* < 0.01; ^*∗∗∗*^*p* < 0.001.

## Data Availability

The data of this study are not available due to ethical concerns. We must protect patient privacy and security and follow the ethical rules of our institutions and their restrictions on data sharing.
